# Application of RiTiCE in understanding hydro-meteorological controls on ice break-up patterns in River Tornionjoki

**DOI:** 10.1007/s10661-024-12910-w

**Published:** 2024-07-27

**Authors:** Abolfazl Jalali Shahrood, Amirhossein Ahrari, Niina Karjalainen, Björn Klöve, Ali Torabi Haghighi

**Affiliations:** 1 Water, Energy and Environmental Engineering Research Unit, University of Oulu, Pentti Kaiteran katu 1, P.O. Box 8000, 90570 Oulu, Finland; 2 Centre for Economic Development, Transport and the Environment for Lapland, Hallituskatu 3 B, 96101 Rovaniemi, Finland

**Keywords:** River ice, Ice break-up, RiTiCE, Cold climate

## Abstract

The Arctic region experiences significant annual hydrologic events, with the spring flood and ice break-up being the most prominent. River ice break-up, in particular, poses high socioeconomic and ecological expenses, including morphological changes and damage to riverine structures. This study aims to investigate the spatiotemporal patterns of river ice in the River Tornionjoki, including the timing of ice break-up at different latitudes. We utilized observation data and remote sensing techniques to track changes in ice patterns overtime on the River Tornionjoki. The study indicates that the ice break-up in the River Tornionjoki basin typically occurs during Apr-Jun based on the reach location in different latitudes; therefore, different stations behave according to their latitudinal location. We observed significant spatial variations in ice break-up timing across the basin, with an earlier break-up in the lower latitudes compared to the upper latitudes. The average ice break-up day in lower latitude stations ranges between 200–205, while in higher latitude stations the average ice break-up day ranges between 215–228.

## Introduction

The Arctic region's most significant annual hydrologic event is the spring flood and the ice break-up phenomena (Pavelsky & Smith, 2004; Rouse et al., 1997). River ice break-up is a significant event that is accompanied by high socioeconomic expenses (Prowse et al., 2011) as well as ecological (Prowse & Culp, 2003; Scrimgeour et al., 1994) and morphological changes (de Rham et al., 2008; Uunila & Church, 2014). Ice break-up causes annual damages of more than $100 and $60 million in the United States (White & Eames, 1999) and Canada (Beltaos, 2000), respectively. Flooding due to ice jams is responsible for most of this damage, affecting bridges, dams, hydropower equipment, and river-side structures (Ettema et al., 1999). The 2001 break-up season in Russia cost more than $120 million (USD) (Brakenridge et al., 2001).

The break-up phase is a significant erosive event typically occurring during the year's peak flow (Gray & Prowse, 1993; Prowse, 2001). Along with several other erosive and depositional characteristics, ice movement during a break-up is linked to significant bank erosion and temporary or permanent flow diversion into secondary channels (Prowse, 2001). River ice break-up is acknowledged as a significant morphological factor that can cause order-of-magnitude increases in suspended sediment concentrations (Beltaos et al., 1994; Gray & Prowse, 1993). From the biological perspective, ice break-up is crucial to the ecology of the riverine system (Scrimgeour et al., 1994; Zhang et al., 2020). For many perched lakes next to Arctic rivers and delta habitats near their mouths, spring break-up flooding is the only source of recharge (Marsh & Lesack, 1996; Prowse & Conly, 1998; Prowse & Lalonde, 1996). Similarly, considerable sediment and carbon exchange between the river and its floodplain can frequently only occur during the ice break-up (Smith & Alsdorf, 1998). The location and timing of ice break-up have been the subject of several studies (e.g., T. D. Prowse & Conly, 1998; Beltaos, 1990, 1997). The disruption to fish breeding sites, and flooding of freshwater riparian ecosystems, including deltas, are some of the effects of ice break-up (Cameron & Lambert, 1971). The spring break-up events for northern regions cause significant problems for northern transportation networks and raise expenditures for local governments, businesses, and lives (USACE, 2002).

Several factors, including the form of the hydrograph, ice thickness, channel curvature, channel width, cumulative degree days of warming, and snowpack depth in basins, may all affect the time and location of break-up (Pavelsky & Smith, 2004). The geographical setting, including heat exchange, wind, precipitation, latitude, and altitude, as well as the waterbody's morphometry and heat storage capacity govern ice phenology (Jeffries & Morris, 2007; Korhonen, 2006; Williams & Stefan, 2006). However, air temperature is the predominant component influencing ice phenology (Ionita et al., 2018). The link between Eurasian air temperatures and the river ice cover is strong despite its complexity (Ruosteenoja, 1986; Williams, 1971; Williams & Stefan, 2006). On the other hand, preceding surface air temperatures provide a seasonal energy flux that is well correlated with ice breakup and freeze-up. As a result, modifications to river ice cover might be interpreted as modifications to the climate-driving factors. Changes in ice breakup and freeze-up dates provide another data source for studying climate patterns (Newton & Mullan, 2021).

Although ice break-up events are widely understood, there have not been many attempts to characterize their spatial and temporal patterns at the basin scale (de Rham et al., 2008). The freeze-up and breakup of lake and river ice is a major control on hydrological and biogeochemical processes at high latitudes (Newton & Mullan, 2021). Over the last century, ice phenology has become increasingly important for energy and water balances (Rouse et al., 2003; Weyhenmeyer et al., 2011), as well as infrastructure such as ice roads (Mullan et al., 2017). On the other hand, understanding the river ice phenology at the basin scale is particularly important for predicting and managing potential risks associated with flooding, ice jams, and infrastructure damage.

Newton and Mullan (2021) studied the river ice phenology during 1931–2005 in the Northern Hemisphere using the Global Lake and River Ice Phenology Database from the National Snow and Ice Data Center (NSIDC). The database includes the freeze-up and break-up dates of 865 Northern Hemisphere stations. The break-up dates are defined as the “the date of the last ice breakup before the open-water season” (Newton & Mullan, 2021). The database has specific definitions for break-up/freeze-up events that may vary between different sites. In another attempt, de Rham et al. (2008) found the temporal variations of river ice break-up in Mackenzie River Basin using the pen-recorded charts, station description, hydrometric survey notes, gauge and benchmark history, discharge measurement tables, station analysis and annual water-level tables. Prowse and Bonsal (2004) studied the river ice break-up trends using historical records. Jiang et al., (2008) investigated the long-term changes in the ice phenology of the Yellow River using recorded datasets. Šarauskienė and Jurgelėnaitė (2008) researched the impact of climate change on ice phenology in 13 stations in Lithuania using the in-situ data. Pavelsky and Smith (2004) found the spatiotemporal patterns of river ice break-up in the Ob, Lena, Yenisey, and Mackenzie Rivers using the datasets of MODIS and AVHRR imagery. Additionally, Cooley and Pavelsky (2016) developed the automated ice detection tool using the MODIS imagery to find the break-up patterns in Arctic and Beaton et al. (2019) found the break-up timing using MODIS in Northern Ontario. For such wider channels, remote sensing could be a proper solution to find the patterns, however, in the narrower rivers the spatial resolution of the remote sensing datasets is not enough to distinguish the ice/water from the surrounding environment. River-ice condition records are regularly gathered in many cold-region countries, albeit by numerous agencies and for diverse purposes. Consequently, the types and methods of observations lack uniformity, leading to variations in the length and quality of the recorded data (Prowse et al., 2007).

Our current study aims to provide a unique definition for the break-up event which is considered to be consistent at any location using the annual daily hydrographs. Consequently, this study fills the data gap of ice break-up events which are not recorded in some locations and addresses the gap on narrower reaches where the remote sensing datasets are not suitable to use. This study investigates the ice phenology of the River Tornionjoki basin, including the timing of ice break-up patterns in different latitudes. The study utilizes observation data and remote sensing techniques to track changes in ice patterns over time. We aim to provide a better understanding of the dynamics of river ice cover at the river scale.

## Materials and methods

### Study area

River Tornionjoki with a catchment area of 40,157 km^2^ and mean annual flow of 400 m^3^.s^−1^ is a transboundary river between Sweden and Finland, and it is a pristine riverine system which stretches for 520 km from Swedish Lapland, where the Torneträsk lake is located. It drains into the Gulf of Bothnia, the northernmost rim of the Baltic Sea (Jalali Shahrood et al., 2023; Romakkaniemi, 2008). River Tornionjoki is the world's most productive river for Atlantic salmon. The river system is the source of half of the wild salmon in the Baltic Sea (Huusko et al., 2020).

The spring flood peak, typically brought on by snow and ice melting, happens between mid-May and mid-June. River flow drops after the spring flood and often remains below 600 m^3^s^−1^ during the summer. Autumn flow conditions are more unpredictable and frequently have a second flow peak after the rainy season. August marks a rapid drop in water temperature, and October is often when the ice cover appears. Due to the water being confined beneath the ice cover, river flow, and temperature are typically low and stable during the winter (Huusko et al., 2020).

The geography of the Torneträsk area is highly variable, with elevations varying from 342 to 1900 m above sea level (Andersson et al., 1996). The Torneträsk region is characterized by a distinct northwest-southeast oceanic-continental gradient, with precipitation and winter temperatures gradually decreasing as one moves eastward from the Atlantic Ocean and the influence of the Scandes Mountains' rain shadow (Pascual, 2022). Previous studies showed that the average annual air temperature at the Abisko Scientific Research Station (385 m a.s.l.) increased by 2.5 °C between 1913 and 2006 (Callaghan et al., 2010). This region has total annual precipitation ranging from > 1000 mm in the northwest to 300 mm in the center and southeastern sections. The mean annual precipitation increased from 301 mm in 1961–1990 to 357 mm in 2010–2019 (Pascual, 2022). This area can be divided into two regions based on relief: (1) a premontane region in the east and (2) a montane region in the west. Many peaks in the mountainous area are more than 1,000 m above sea level (m a.s.l.). The premontane region is a low relief area with a range of 300 to 600 m above sea level and is composed of vast plains with lingering hills (Lidmar-Bergström, 1995).

**Fig. 1:**
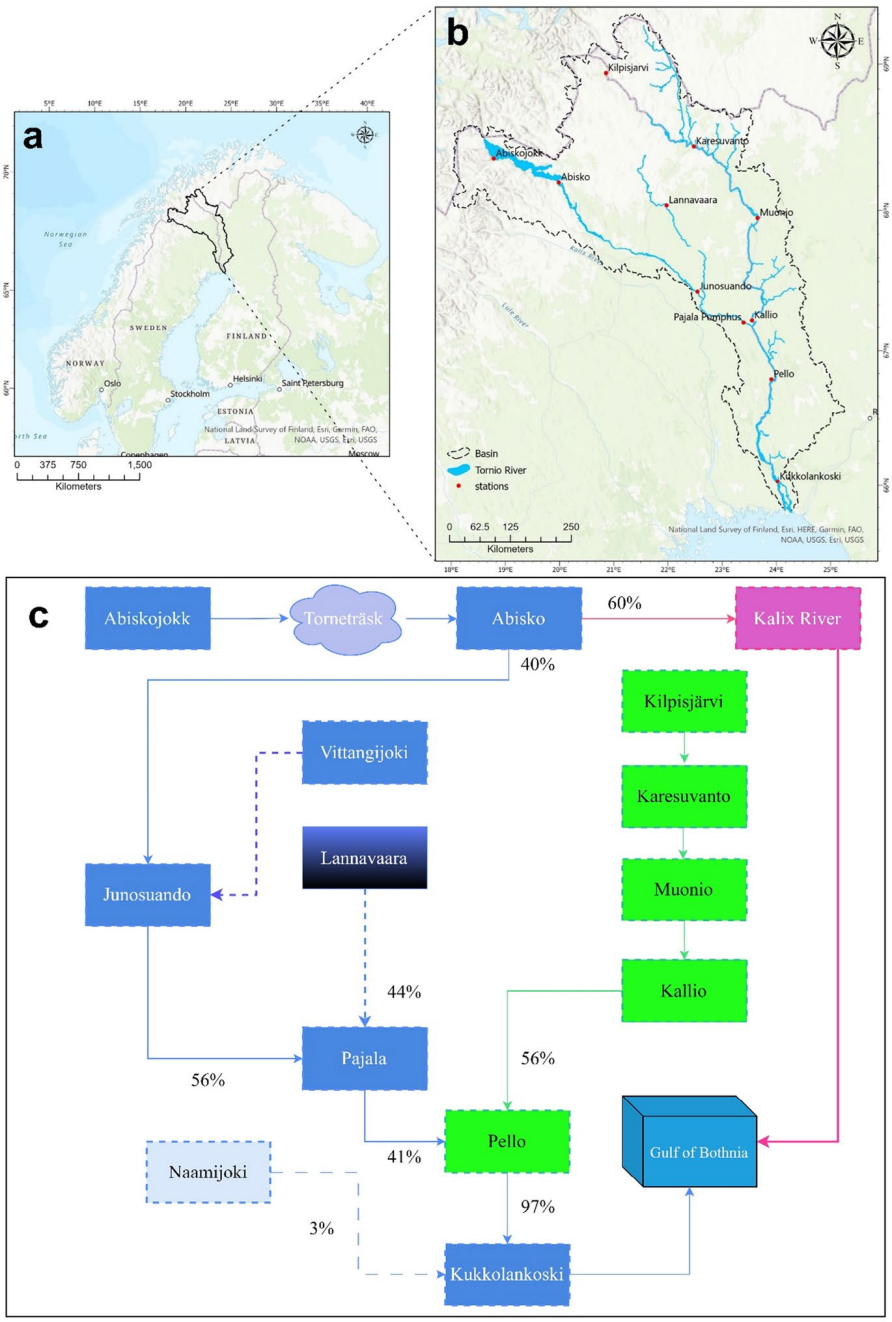
**a** Location of River Tornionjoki basin in Finland and Sweden, **b** River Tornionjoki basin and all 11 gauging stations along the river, **c** Schematic diagram of River Tornionjoki and the connections of all stations together, the percentages show the share of flow from each tributary and at the confluence points

The study area is in the northernmost part of Sweden and Finland, including several stations along the River Tornionjoki and Muonio River. The Muonio River is the largest tributary of the River Tornionjoki, and it originates in the northern part of Sweden and Finland and flows on the border for approximately 140 km before joining the River Tornionjoki at Kallio (Fig. 1).

In this study we used daily discharge data (for river ice break-up day estimation, i.e., BUD), Land surface temperature (TS) from MODIS LST product, Longwave (LW) and Shortwave (SW) irradiance, Relative Humidity (RH), Wind speed (WS), and Air Temperature at 2 m (T2M) for the period of 2002–2020 on 11 stations along the River Tornionjoki and two tributaries, Muonio and Lannavaara (as depicted in Fig. 1). Of these, six stations were located along the main corridor of the River Tornionjoki, including Abiskojokk (68.36◦N, 18.78◦E), Abisko (68.19◦N, 19.98◦E), Junosuando (67.42◦N, 22.54◦E), and Pajala (67.20◦N, 23.39◦E), all located above the confluence point with the Muonio River. The remaining two stations, Pello (66.79◦N, 23.90◦E) and Kukkolankoski (66.03◦N, 24.02◦E), were situated downstream of this confluence point. Almost 60% of the flow is bifurcated from the River Tornionjoki as the Kalix River, upstream of the Junosuando station. Regarding the Muonio River, we analyzed daily flow data from Kilpisjärvi (68.94◦N, 20.85◦E), Karesuvanto (68.44◦N, 22.47◦E), Muonio (67.94◦N, 23.65◦E), and Kallio (67.2◦N, 23.55◦E) stations. Furthermore, Lannavaara (68.03◦N, 21.97◦E) station, which is located on a tributary that joins the River Tornionjoki between Junosuando and Pajala, was also selected for analysis. It is worth mentioning that two tributaries, Vittangijoki and Naamijoki, are excluded from this study due to lack of data for Vittangjoki and minor contribution in the main river flow (i.e., 3% of flow) for Naamijoki (Fig. 1).

Latitude wise, the selected stations are divided into three categories of (1) relatively **high-latitude stations** between 67.9^◦^N and 69.0^◦^N, including Abiskojokk, Abisko, Lannavaara, Kilpisjärvi, Karesuvanto, and Muonio (2) relatively **mid-latitude stations** between 66.79^◦^N and 67.43^◦^N, including Junosuando, Pajala, Kallio and Pello, and (3) relatively **low-latitude station** as Kukkolankoski (Fig. 1). A schematic diagram showing the connections between stations and the whole river network is depicted in Fig. 1.

### Data source

The flow data were obtained from Swedish Meteorological and Hydrological Institute (SMHI, 2024) and Finnish open hydrology data (SYKE, 2024). Surface temperature (TS) data were obtained from MODIS LST product in Google Earth Engine. Longwave irradiance (LW), Shortwave irradiance (SW), Relative Humidity (RH), Air temperature at 2m (T2M), and Wind Speed (WS) data were collected from NASA—Prediction of Worldwide Energy Resource data access viewer (POWER, 2024).

This study focuses on further enhancing the RiTiCE (River Timing Characteristics and Extremes) tool developed earlier by (Jalali Shahrood et al., 2023). The objective of RiTiCE is to quantify and detect the Phase Change Timing (i.e., PCT) on hydro-meteorological variables. It estimates the timing of Break-Up Day (i.e., BUD) using the flow data, the onset of warming using the temperature data and detects the PCT in other parameters mentioned earlier. Previously RiTiCE BUDs were verified by observed ice break-up records on Kukkolankoski station with a correlation coefficient of 0.8 (Jalali Shahrood et al., 2023). To further verify the BUD values and also to visualize ice patterns in River Tornionjoki, raw Sentinel-1 data were employed from 2016 to 2020.

### Methods

This study covers different analyses of hydro-meteorological and RS data. Each analysis is explained in detail in the following sections. The daily discharge data is used to determine the ice BUD in River Tornionjoki and to spot locations where the river ice breaks up later or sooner than other stations. The daily temperature time series from MODIS gap-filled and assimilated data is used to find the interconnection between the surface temperature and BUDs. Finally, the Sentinel -1 SAR data are used to visualize the break-up events estimated by RiTiCE, as explained below.

#### RiTiCE – break-up detection tool

RiTiCE requires annual daily records in order to determine the PCT. The dataset's structure must conform to following guidelines: (1) The dataset needs to be adjusted for leap years by removing 29 February, ensuring that all years have a consistent 365-day duration. (2) The dataset should be organized according to the water year (or hydrological year), which begins on 1 October and ends on 30 September in each year. In order to estimate the PCT of different variables, we have created the RiTiCE detection module tailored to each individual variable. Regarding discharge, the PCT refers explicitly to the timing of the river ice break-up day which is defined as the date at which the flow hydrograph starts to rise in springtime right after the long period of low flow in winter—which is usually during April-June depending on the case study-. this definition is different from break-up date which is defined as “The date on which a body of water is first observed to be entirely clear of ice, and remains clear thereafter” (IAHR, 1980). The choice of the PCT detection method is based on the annual time series shape and pattern of each parameter. Table 1 displays the PCT detection methods corresponding to each parameter.

**Table 1:** Selection of PCT detection method based on the nature and annual pattern of each data. DVD (Daily Value Difference, MeRCAT (Median-Referenced Cumulative Area Transition), ZCAT (Zero-Referenced Cumulative Area Transition)

Parameter	PCT detection Method
BUD	DVD
LW	MeRCAT
RH	MeRCAT
SW	DVD
T2M	ZCAT
TS	ZCAT
WS	MeRCAT

Daily Value Difference (DVD) has been previously tested on other case studies (Jalali Shahrood et al., 2023) to find the river ice BUD. Zero-Referenced Cumulative Area Transition (ZCAT) was tested on assimilated daily surface temperature data -previously known as Temperature Transition Point (or TTP)- (Jalali Shahrood et al., 2023), and Median-Referenced Cumulative Area Transition (MeRCAT) is a modification of ZCAT to apply on datasets that change their phase over their median value.

RiTiCE was initially developed in MATLAB. The break-up detection tool in RiTiCE detects the BUD based on the annual daily hydrograph. RiTiCE uses the Daily Value Difference (DVD) between each pair of consecutive discharge data to detect the BUD on each annual daily hydrograph:

1 $${\Delta }_{i}=\frac{{Q}_{i+1}-{Q}_{i}}{T}$$

where, Δ_i_ is the “differenced” value, Q is the discharge value, and T is the time which equals 1 as RiTiCE uses a daily hydrograph. Using the results from the series of differences, we observe that the point at which the discharge starts to rise in the spring has nearly equal values around 0. This happens as the discharge time series follows a line with a gradient of around zero during winter. Therefore, the last value ending the consecutive zero gradient pair of points is considered as BUD.

#### MODIS Gap-filled land surface temperature

To assess the relationship between ice temperature and break-up events, daily Land Surface Temperature (LST) is needed. While the daily LST are already provided by MOD11A1 globally, mostly cloudy sky conditions in high latitude regions caused many gaps in satellite observations. To deal with the existing gaps, we used the data assimilation technique through Temporal Fourier Analysis (TFA), where the MODIS LST data gaps were filled out by combination with ERA5 skin temperature product (Scharlemann et al., 2008; Shiff et al., 2021). The TFA method recovered missed pixels by adding temperature anomaly to the MOD11A1 to produce continuous non-cloudy LST time series. TFA describes the seasonal temperature cycle in terms of annual, bi-annual, and tri-annual harmonics based on amplitude, phase, and frequency characteristics. TFA reconstructs a smooth temperature series for both (MODIS and ERA5) LST products, which are considered as climatological expected temperatures:

2 $${LST}_{clim}(t)=\overline{LST }+\sum\nolimits_{i=1}^{n}{A}_{i}\text{cos}({\omega }_{t}-{\varphi }_{i})$$

where ${LST}_{clim}(t)$ is the climatological MODIS LST at Julian day t; $\overline{LST }$ is the mean annual LST, ${A}_{i}$ is the amplitude of i^th^ harmonic, while n is the number of harmonic components (3 harmonics selected as annual, bi-annual, and tri-annual). ${\varphi }_{i}$ is the phase and ${\omega }_{t}$ is frequency (${\omega }_{i}=2\pi i/365$) of the i^th^ harmonic component. To calculate ${A}_{i}$ and ${\varphi }_{i}$, time series of one year (365 Julian days) with the 2002–2019 mean clear sky LST data for each Julian day also were used.

**Fig. 2:**
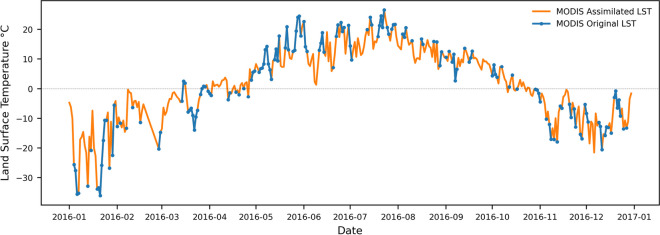
MODIS assimilated land surface temperature vs. MODIS original land surface temperature

**Fig. 3:**
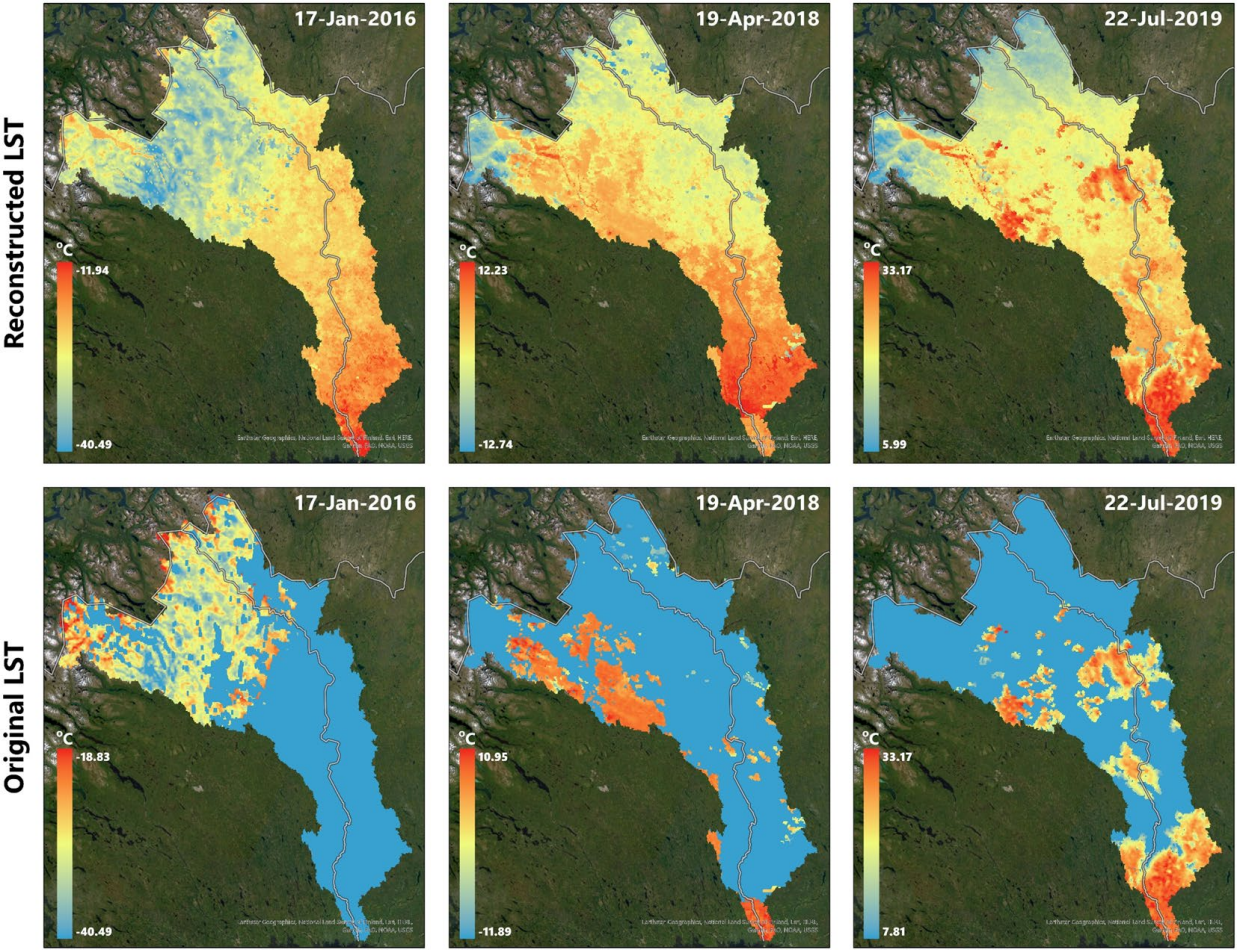
Visualization of the MODIS Assimilated vs. Original LST coverage on River Tornionjoki Basin

The surface temperature at a specific date and time is a function of two complementary components: a) long-term average temperature at that specific date and time (climatology) and b) deviation from that mean due to the weather (anomaly). Therefore, to estimate actual LST (${LST}_{count}\left(t\right)$), LST anomaly from ERA5 product (${T}_{anom}(t)$) added to the fine-scale observed MODIS climatological LST (Figs. 2 and 3):

3 $${T}_{anom}\left(t\right)=T-{T}_{clim}$$

4 $${LST}_{count}\left(t\right)={LST}_{clim}\left(t\right)+{T}_{anom}(t)$$

It is worth noting that the spatial resolution of the MODIS datasets is 1 km, and a single value of temperature is averaged from all pixels covering the area of interest (i.e., each station).

#### Surface temperature phase change timing detection

**Fig. 4:**
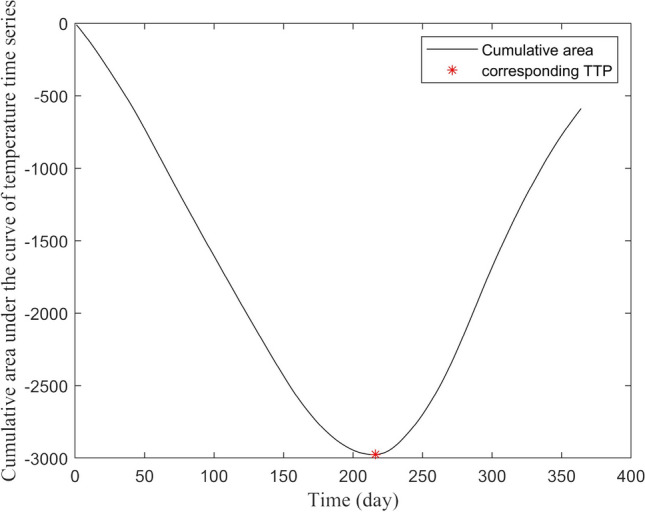
Absolute Minimum value of the cumulative area under the temperature time series showing the least difference between positive and negative phase in Abisko station in 2003–2004 (i.e., TS PCT)

The fluctuation of daily temperatures throughout the year follows a distinct seasonal pattern, with negative values predominating in winter and positive values in summer. The temperature data exhibits stochastic behavior, meaning that it fluctuates unpredictably over time due to various factors such as weather patterns, atmospheric conditions, and other environmental factors. The Zero-Referenced Cumulative Area Transition (ZCAT) was determined in each station by analyzing the transition patterns from the negative to positive temperature phase. The ZCAT was found by calculating the cumulative area under the curve of the temperature time series, where the cumulative area graph reaches its absolute extreme value (min or max depending on the variable). This method identifies the point at which the cumulative positive and negative values are the least different (Fig. 4). PCT was assessed on all variables and on all 11 stations for 18 years, and the results are shown in summary as box-plot, and correlation heatmaps.

#### Sentinel -1 visualization data

Sentiel-1 GRD products were used to detect ice cover on the river in the study area as SAR sensors provide high spatial resolution microwave imagery with transparency to the atmosphere impacts and cloud cover. Since Sentinel-1 products are available in the Google Earth Engine platform geometrically corrected, only radiometric calibration is performed to obtain sigma0, and then to reduce the speckle effects, speckle filtering is also implemented. To reduce riverside landcover impacts on the water backscatters, non-river land covers were masked from each image, and then multi-temporal radar backscatters were compared by weekly frequency. It is worth noting that the spatial resolution of the Sentinel-1 datasets is 30 m after applying the speckle filtering and the VV polarization is used so that more energy is received by the sensor and anomalies can be shown better.

## Results

### Correlation heatmap between different variables and BUD

**Fig. 5:**
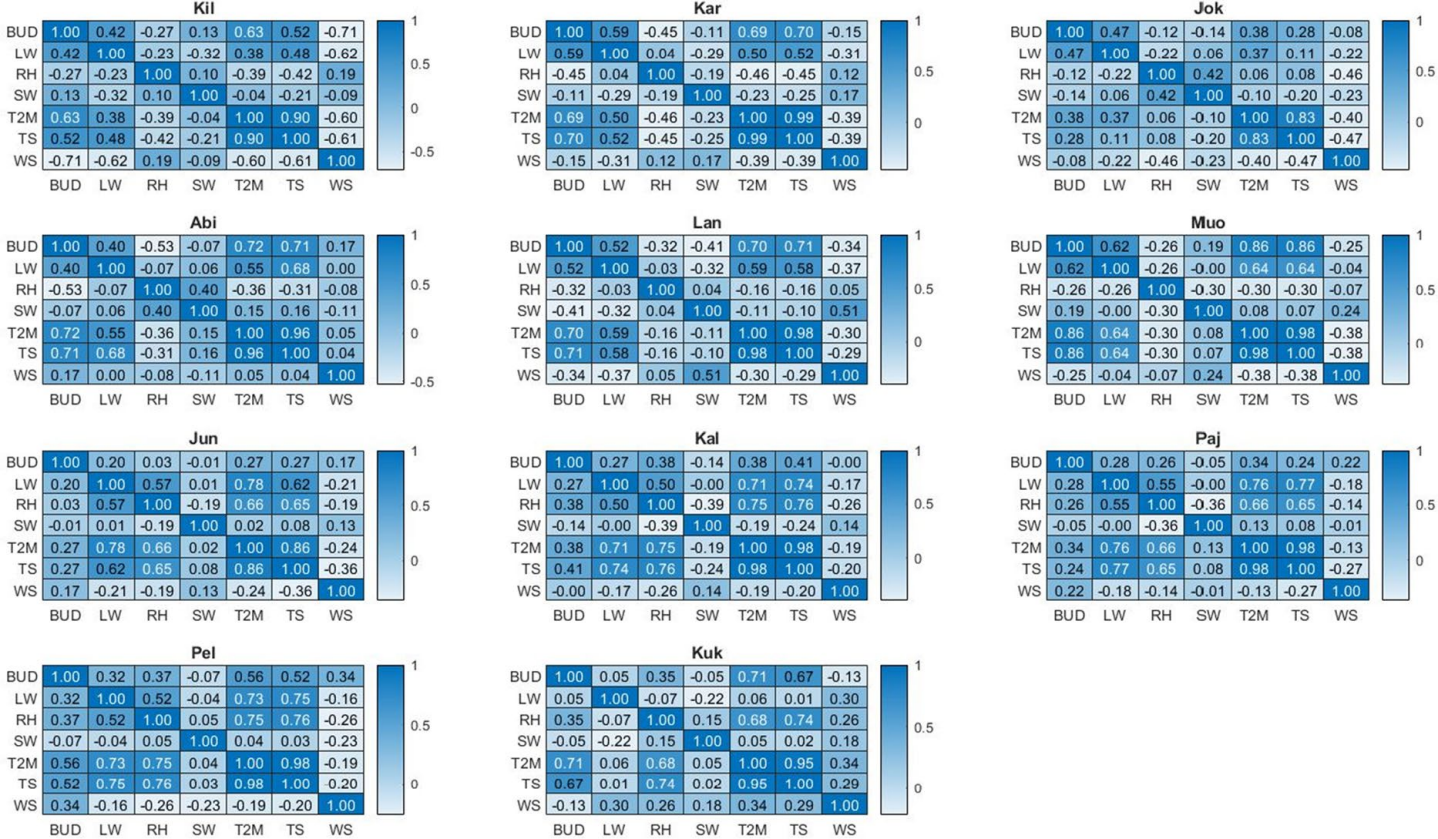
Correlation heatmaps of different variables in 11 stations along Tornionjoki. Stations are sorted from higher latitude to lower latitude and abbreviated as Kilpisjärvi (Kil), Karesuvanto (Kar), Abiskojokk (jok), Abisko (Abi), Lannavaara (Lan), Muonio (Muo), Junosuando (Jun), Kallio (Kal), Pajala (Paj), Pello (Pel), and Kukkolankoski (Kuk)

The correlation heatmap on 11 stations showing the relationship between different variables’ PCT and BUD indicates that in Kil, Kar, Abi, Lan, Muo, Pel, and Kuk stations, the TS, and T2M variables have the most correlation with BUD. In high-latitude stations, the LW variable has moderate correlation with BUD, while in mid-latitude and Low-latitude stations the LW does not show significant correlation with BUD (Fig. 5).

### Summarized results of PCT in variables

**Fig. 6:**
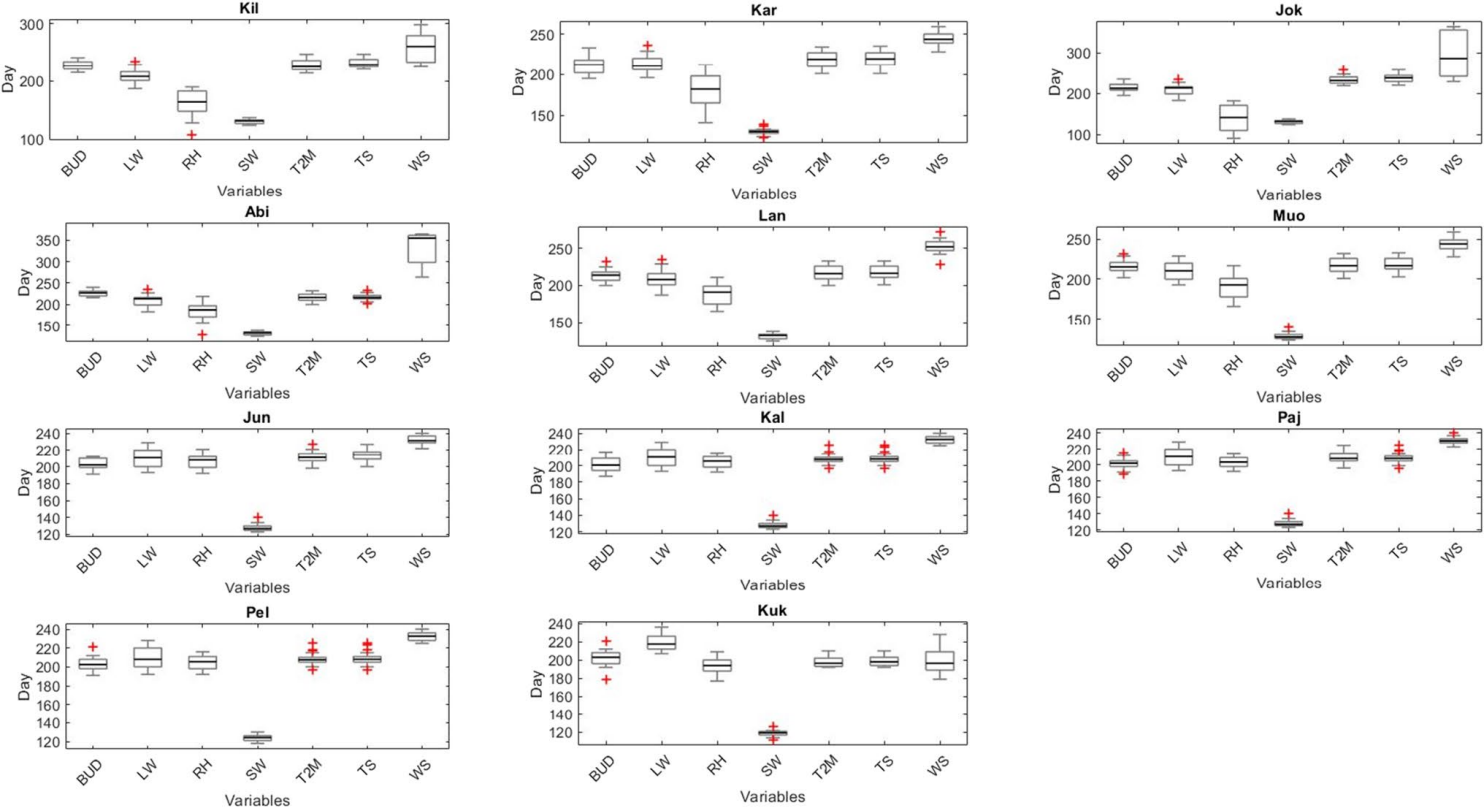
Summary of PCT in different variables in 11 stations along Tornionjoki. Stations are sorted from higher latitude to lower latitude and abbreviated as Kilpisjärvi (Kil), Karesuvanto (Kar), Abiskojokk (jok), Abisko (Abi), Lannavaara (Lan), Muonio (Muo), Junosuando (Jun), Kallio (Kal), Pajala (Paj), Pello (Pel), and Kukkolankoski (Kuk)

The boxplot provided in Fig. 6 reveals the summarized PCT of different variables showing the SW changing phase earlier and WS later than the other variables. LW, BUD, T2M, TS, and in some stations, RH change their phase about the same time as each other. In high-latitude stations the RH PCT occurs earlier than the BUD, while in low and mid-latitude stations this happens almost at the same time with BUD.

### Spatiotemporal variation of BUD in River Tornionjoki

**Fig. 7:**
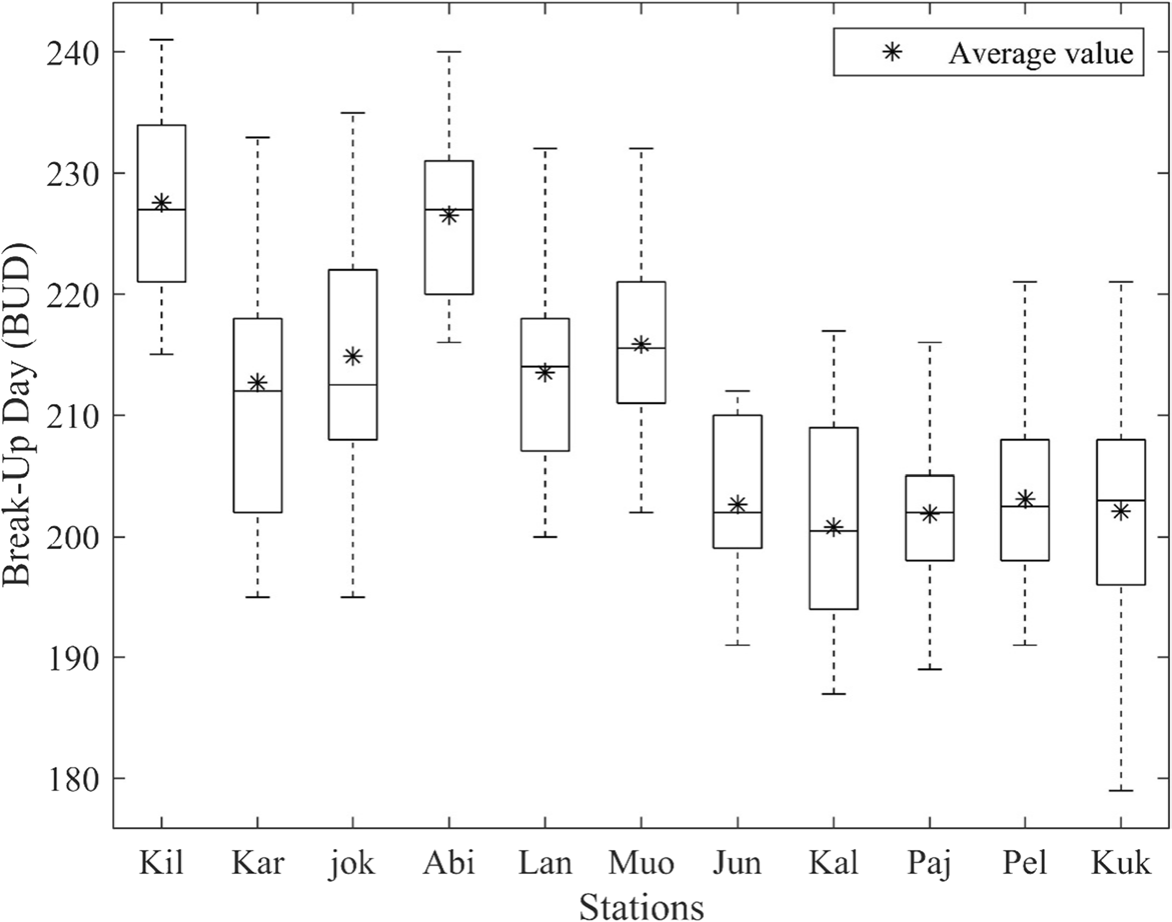
BUD variation in different stations along Tornionjoki. Stations are sorted from higher latitude to lower latitude and abbreviated as Kilpisjärvi (Kil), Karesuvanto (Kar), Abiskojokk (jok), Abisko (Abi), Lannavaara (Lan), Muonio (Muo), Junosuando (Jun), Kallio (Kal), Pajala (Paj), Pello (Pel), and Kukkolankoski (Kuk)

According to the findings presented in Fig. 7, the break-up of river ice in low and mid-latitude stations (such as Pello, Kukkolankoski, Pajala, Kallio, and Junosuando) occurs earlier than in high-latitude stations, including Abiskojokk, Abisko, Karesuvanto, Kilpijärvi, Muonio, and Lannavaara. The trend shows that the river ice tends to melt later as the latitude increases. Moreover, the break-up dates in 18 years display more variability in Karesuvanto, Kallio, Kukkolankoski, and Abiskojokk compared to other stations, indicating that the break-up pattern in these locations is less consistent, and their BUD values cover a wider range. The BUD in Abisko is skewed to the right, revealing that later BUD values are closer together than the earlier BUD events in that station. In contrast, BUD values in Junosuando are skewed to the left; it depicts that earlier BUD values are closer to each other than the later BUD values. Kilpisjärvi and Abisko have the highest median and average BUD values compared to other stations. All mid and low-latitude stations have almost the same median and average BUD values.

**Fig. 8:**
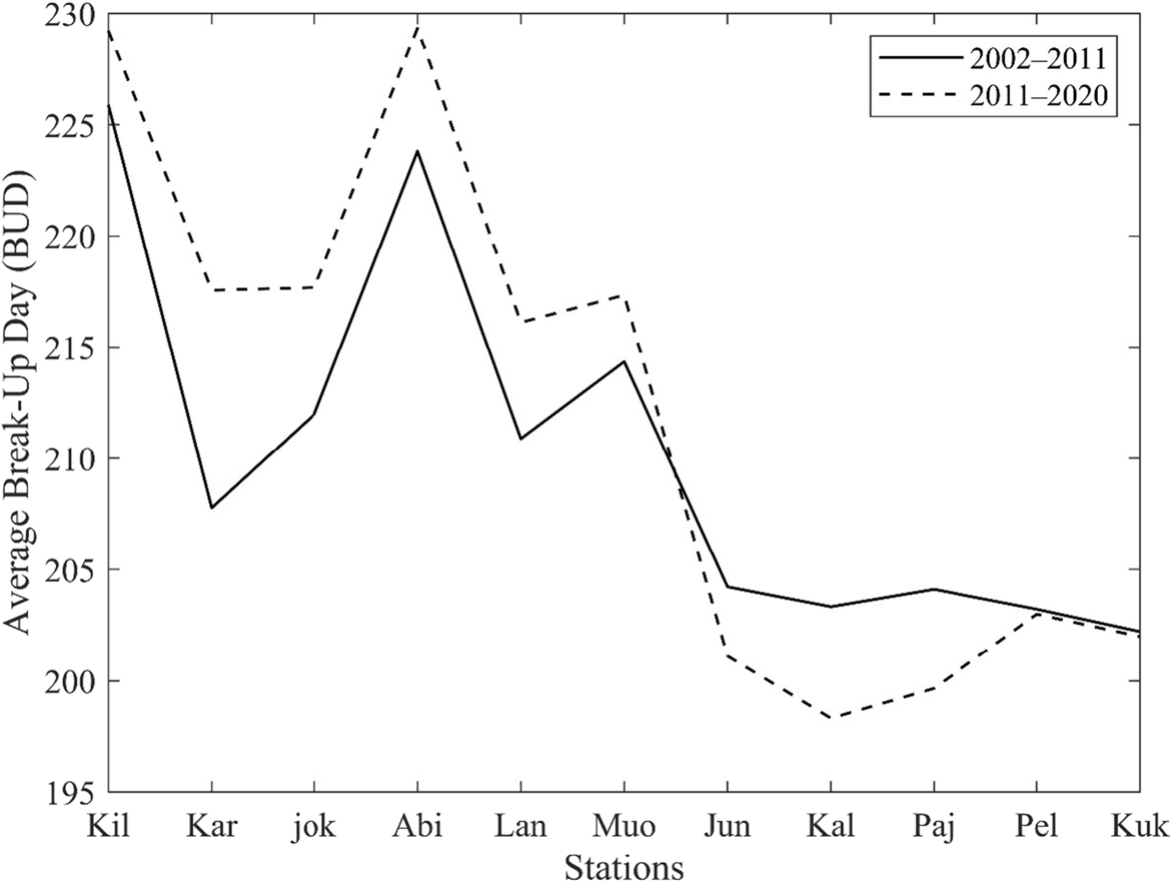
Nine-year partitioning of BUD along River Tornionjoki. Stations are sorted from higher latitude to lower latitude and abbreviated as Kilpisjärvi (Kil), Karesuvanto (Kar), Abiskojokk (jok), Abisko (Abi), Lannavaara (Lan), Muonio (Muo), Junosuando (Jun), Kallio (Kal), Pajala (Paj), Pello (Pel), and Kukkolankoski (Kuk)

Figure 8 compares the BUD patterns in two 9-year periods, revealing changes in BUD over time. The results show that high-latitude stations experienced later break-up events in the second period, whereas mid and low-latitude stations experienced earlier break-up patterns. Upon closer examination of the high-latitude stations, including Abiskojokk, Abisko, Karesuvanto, Lannavaara, Muonio, and Kilpisjärvi, an average shift of 6, 6, 10, 5, 3, and 3 days, respectively, was observed in their BUD. These stations experienced a delayed break-up, implying that the rivers in these regions were frozen for a longer duration during the second period compared to the first period.

Conversely, the mid and low-latitude stations, namely Junosuando, Kallio, Pajala, Kukkolankoski, and Pello, exhibited a different pattern. These stations displayed an average shift in BUD of 3, 5, 4, 0, and 0 days, respectively. The break-up events at these stations occurred earlier during the second period, indicating a shorter duration of river ice cover in these regions.

### Surface temperature PCT in River Tornionjoki

**Fig. 9:**
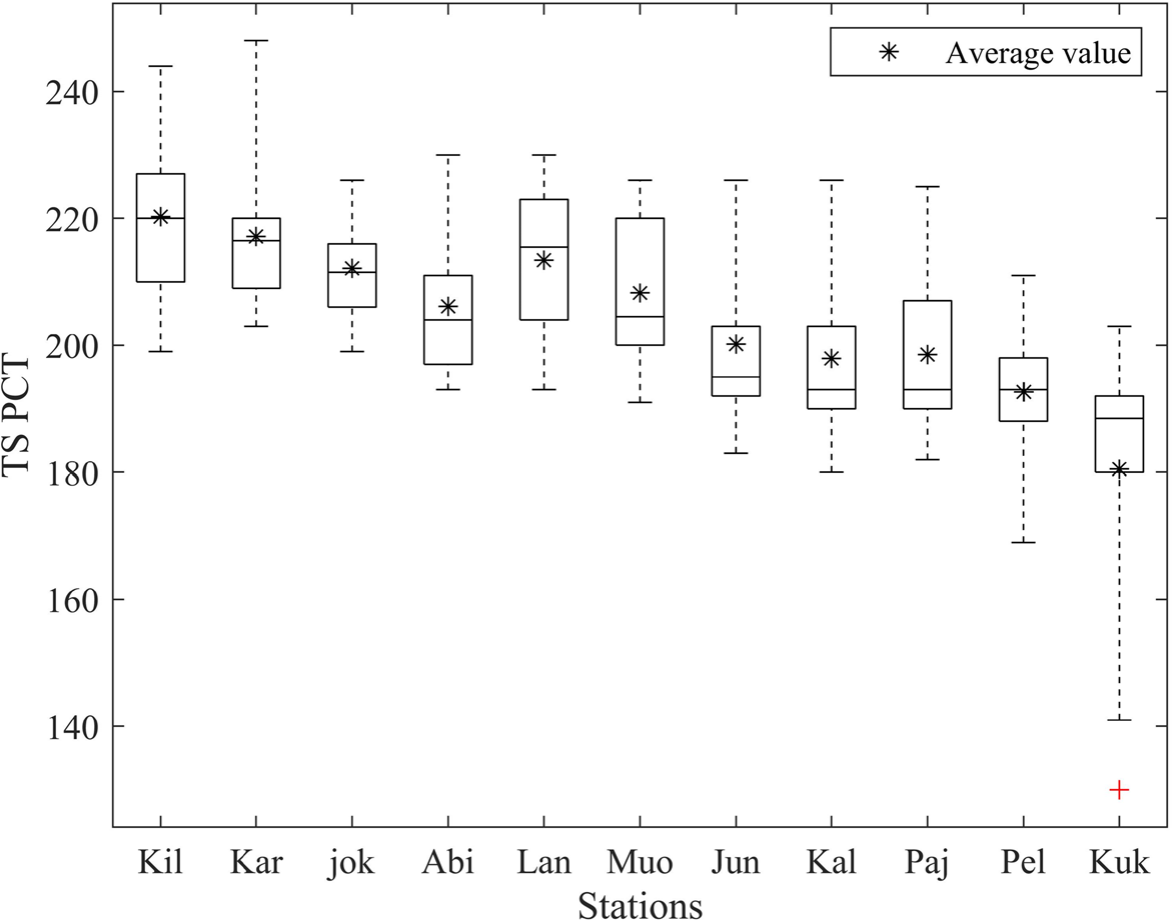
TS PCT variation in different stations along Tornionjoki. Stations are sorted from higher latitude to lower latitude and abbreviated as Kilpisjärvi (Kil), Karesuvanto (Kar), Abiskojokk (jok), Abisko (Abi), Lannavaara (Lan), Muonio (Muo), Junosuando (Jun), Kallio (Kal), Pajala (Paj), Pello (Pel), and Kukkolankoski (Kuk)

As the most dominant factor related to BUD is the temperature, the analysis of PCT in River Tornionjoki (Fig. 9) reveals that, on average, the positive phase in high latitude stations, such as Abiskojokk, Abisko, Karesuvanto, Kilpisjärvi, Muonio, and Lannavaara, starts later than in the mid and low latitude stations. Furthermore, the TS PCT data in Karesuvanto, Junosuando, Kallio, Pajala, Kilpisjärvi, and Kukkolankoski show more dispersed results, indicating that these stations experience less consistent patterns and cover a broader range of TS PCT values over the 18 years, compared to other stations. There is a right skewness in some stations, including but not limited to Karesuvanto, Lannavaara, and Kukkolankoski, which means that the later TS PCT results are closer together. On the other hand, in Junosuando, Kallio, and Pajala, the skeweness is to the left, which reveals that the earlier TS PCT results are closer to each other. Regarding the average TS PCT values, they vary in the range of 180–220.

**Fig. 10:**
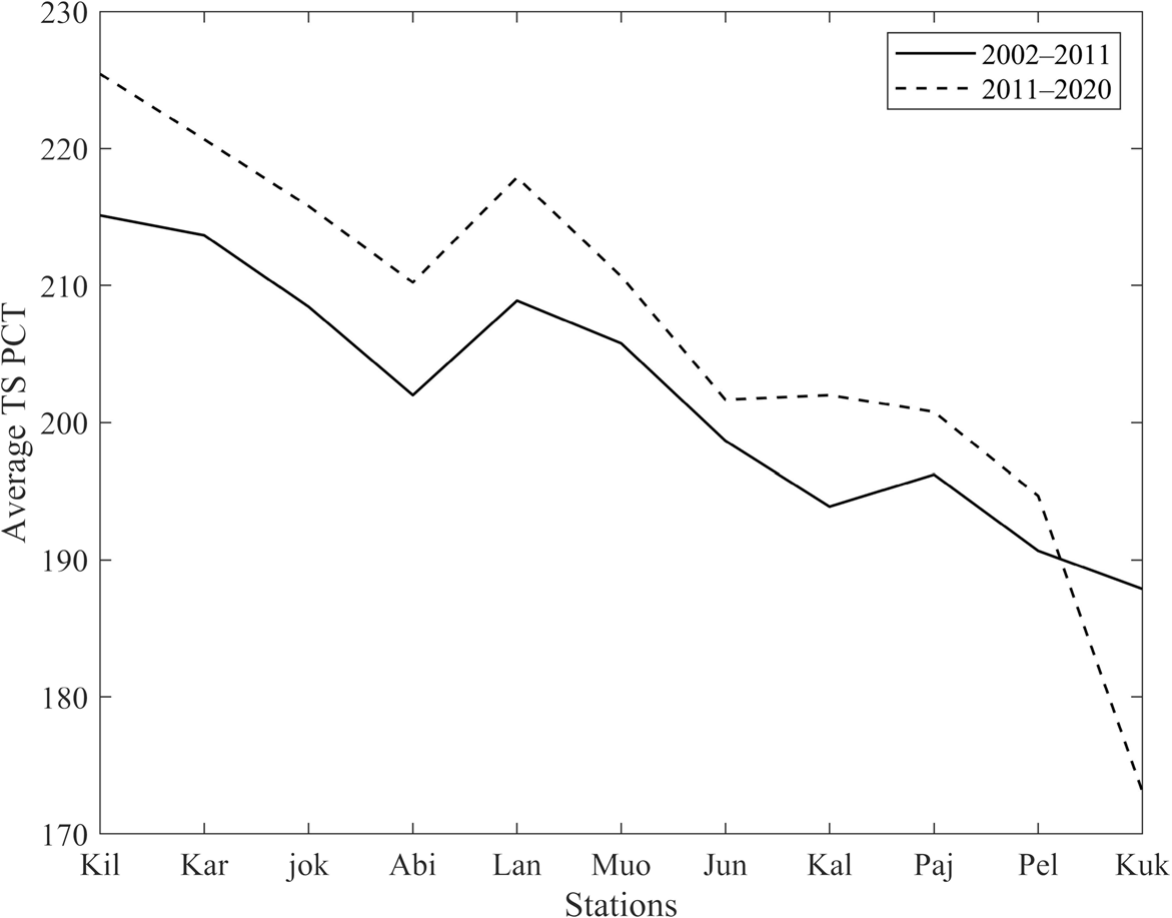
Nine-year partitioning of TS PCT in River Tornionjoki. Stations are sorted from higher latitude to lower latitude and abbreviated as Kilpisjärvi (Kil), Karesuvanto (Kar), Abiskojokk (jok), Abisko (Abi), Lannavaara (Lan), Muonio (Muo), Junosuando (Jun), Kallio (Kal), Pajala (Paj), Pello (Pel), and Kukkolankoski (Kuk)

Figure 10 illustrates the temporal changes in the TS PCT at each of the 11 stations over the entire 18-year study period, which was further divided into two equal periods of 9 years each. The TS PCT represents the point at which the temperature transits from the negative phase to the positive phase in spring, indicating the onset of warmer temperatures. Upon analyzing the TS PCT patterns, it is evident that all stations, excluding Kukkolankoski, exhibited a notable shift towards a later transition to the positive phase during the second 9-year period. This shift implies that, on average, these stations experienced a delayed onset of warmer temperatures compared to the first period. The delayed transition to a positive phase in the second period may indicate a longer winter season or a delayed arrival of spring-like conditions. The inconsistency observed at Kukkolankoski, which did not display the exact change in TS PCT, requires further investigation. It is possible that local factors, such as geographical features or microclimatic influences, contributed to the station's unique response to seasonal transitions.

### Relationship between BUD and TS PCT in different stations

**Fig. 11:**
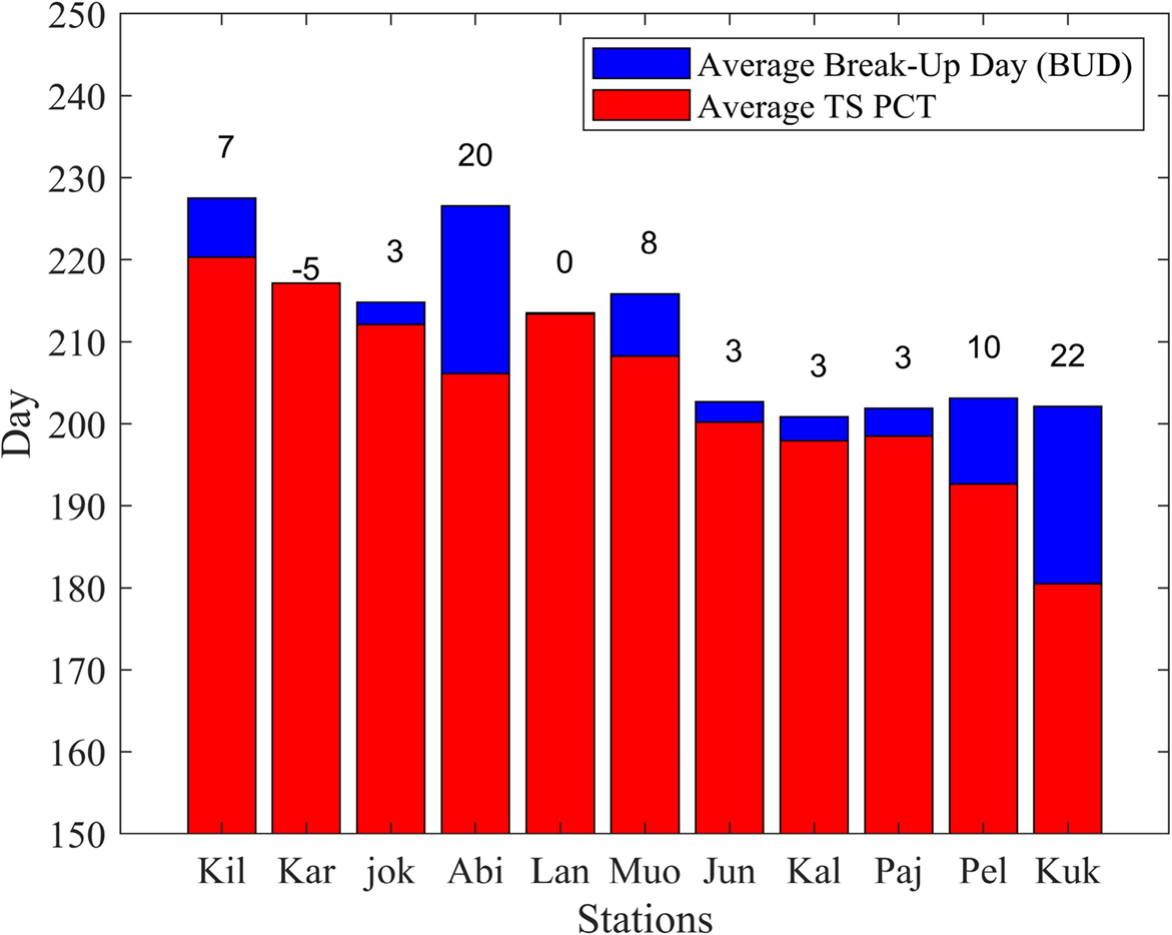
Average BUD (blue bars) and Average TS PCT (red bars) on each station on River Tornionjoki. Stations are sorted from higher latitude to lower latitude and abbreviated as Kilpisjärvi (Kil), Karesuvanto (Kar), Abiskojokk (jok), Abisko (Abi), Lannavaara (Lan), Muonio (Muo), Junosuando (Jun), Kallio (Kal), Pajala (Paj), Pello (Pel), and Kukkolankoski (Kuk), the number on each bar represents the time-lag between the occurrence of TS PCT and the occurrence of BUD

Figure 11 presents a comparison between the average BUD and TS PCT values for each of the 11 stations. The bar chart reveals the similarities in the patterns observed between BUD and TS PCT, providing insights into the relationship between these two variables. The results reveal that the majority of stations exhibit a consistent pattern. In Abiskojokk, Abisko, Lannavaara, Junosuando, Kallio, Pajala, Kukkolankoski, Kilpisjärvi, Muonio, and Pello, the BUD occurs, on average, with a delayed onset relative to the TS PCT. Specifically, these stations experience a 3, 20, 0, 3, 3, 3, 22, 7, 8, and 10-day offset after the TS PCT, respectively. This offset indicates that river ice break-up tends to occur slightly later than the transition to the positive phase of temperature during spring in these locations. The time lag between the TS PCT and BUD suggests that the warming temperatures required for river ice to break up take slightly longer to manifest in these areas. Factors such as regional climate characteristics, geographical features, and hydrological processes could contribute to this observed offset.

However, it is important to note that Karesuvanto exhibits a different pattern than the other stations. In this station, the TS PCT, on average, occurs with a 5-day offset after the BUD. This suggests that, in Karesuvanto, the transition to the positive phase of temperature precedes the break-up of river ice by approximately five days. The reasons behind this unique pattern in Karesuvanto could be attributed to local climatic factors, river dynamics, or other site-specific characteristics that influence the timing of the temperature transition and river ice break-up.

### Tornio ice dynamics

**Fig. 12:**
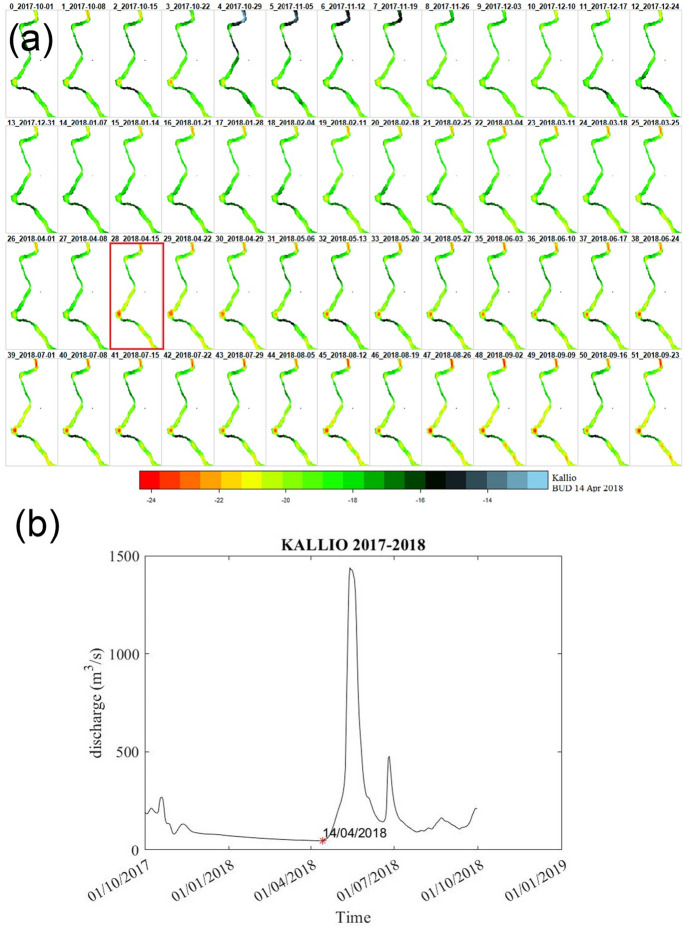
**a** Sentinel -1 SAR imagery visualizes the Kallio station ice break-up event during 2017–2018, the red box shows the right timing of BUD on Sentinel-1 weekly data to compare with the red asterisk in the hydrograph, **b** BUD (red asterisk) on the annual daily hydrograph of Kallio station during 2017–2018

Using Sentinel-1 SAR data, though with a coarse resolution in many stations along River Tornionjoki, enables us to verify the timing of BUD values. In addition, Sentinel-1 SAR data provides information into the spatial and temporal variability of the break-up event at different stations along the river. As an example, in Fig. 12 the average weekly Sentinel-1 dataset at Kallio station is provided for the year 2017–2018. There are 52 weeks showing the river texture dynamics, the color bar shows the backscatter value in Sentinel-1 data in dB, as the surface texture changes (i.e., River turns from ice to water) the backscatter value decreases. Backscatter in the context of SAR data refers to the part of the radar signal that is scattered back to the satellite by the Earth's surface. This backscatter effect is influenced by the surface characteristics such as roughness, moisture content, and geometric structure. The Sigma-0 coefficient (which its values are shown in color bar) represents the radar brightness, which can be related to surface properties. The backscatter value from Sentinel-1 will vary depending on whether a river is frozen or has melted. These variations are due to differences in the physical properties of the surface conditions over which the radar signal is scattered back to the satellite. In Fig. 12, RiTiCE calculated the BUD in Kallio station on 14 Apr 2018, similarly, Sentinel-1 shows the week starting from 15 April 2018 has some texture change in the river (i.e., changing from ice to water). The remaining figures on other stations are inserted in Appendix.

## Discussion

Our analysis across 11 stations along the River Tornionjoki revealed significant correlations between river ice break-up day (BUD) and surface temperature (TS) as well as temperature at 2 m (T2M), particularly in stations like Kilpisjärvi (Kil), Karesuvanto (Kar), and Abisko (Abi). This strong correlation suggests that warmer surface temperatures, indicative of the region’s transition from winter to spring, play a crucial role in the timing of ice break-up (Hallerbäck et al., 2022; Newton et al., 2016; Prowse et al., 2007). This is particularly evident in high-latitude stations where seasonal temperature fluctuations are more pronounced due to the extreme difference in daylight during winter and summer. In terms of irradiance, longwave irradiance (LW) showed a moderate correlation with BUD at high-latitude stations. This could indicate that longwave irradiance, which is largely influenced by cloud cover and atmospheric conditions, may also play a role in the thermal dynamics of river ice. However, this correlation was not evident in mid and low-latitude stations, suggesting that other factors may be more influential in these regions. The lack of significant correlation between BUD and shortwave irradiance (SW) across most stations might be related to the high albedo of ice and snow during the freeze-up season, that reflects most incoming shortwave radiations. Wind speed (WS) also did not show a strong correlation with BUD, which might suggest that its role in ice dynamics is less significant than thermal factors in this study.

The BUD pattern showed a latitudinal gradient in the timing of river ice break-up. This finding is consistent with the hypothesis that higher latitudes experience colder temperatures, which can cause the ice to persist for extended periods. The latitudinal gradient in BUD shows how river ice dynamics react to geographical differences to temperature variations across different geographical locations. Higher latitudes, characterized by prolonged cold periods, exhibit a delayed ice break-up, contributing to the relationship between climate and river ice behavior. The BUD results suggest that the timing of river ice break-up is subject to significant temporal variability, and different latitudes experience distinct changes in BUD patterns. Our results also show that the BUDs in 18 years have more dispersed results in Karesuvanto, Kallio, Kukkolankoski, and Abiskojokk than in other stations. This may be due to the influence of other environmental factors, such as snow cover, local topography, solar radiation, and altitude, that can impact the timing of ice break-up (Novikmec et al., 2013). In Kallio and Abiskojokk the correlation between thermal variables (TS and T2M) are low and not dominating enough as in other stations, in Karesuvanto station the correlation between LW and BUD is almost as significant as thermal variables which could be an influencing factor on its dispersed results in BUD, in Kukkolankoski though, the thermal variables are the only significant driving factors on BUD, and the dispersed results could come from the mechanical ice break-up due to the upstream flow. These findings are important as they approve the impact of climate change on river ice break-up patterns (Li et al., 2023; Prowse & Beltaos, 2002). River ice break-up patterns are an important factor to consider when designing and implementing flood mitigation measures (Lindenschmidt, 2020; Puestow et al., 2004). The results can help municipalities and other stakeholders understand when to anticipate peak river flows and plan accordingly (Lindenschmidt et al., 2015). River ice break-up patterns can also impact navigation and transportation in regions that rely on river transportation (Ettema & Huang, 1990; Shen, 2010). The results can inform decisions on when to open or close navigation channels and adjust transportation schedules accordingly. Areas that experience earlier ice break-up patterns may have a longer period for navigation and transportation, while areas that experience later ice break-up patterns may have a shorter period. These patterns can also have implications for tourism and recreational activities. Areas that experience earlier ice break-up patterns may attract tourists and recreationists for the early spring season, while areas that experience later ice break-up patterns may have a shorter window for these activities.

Our findings on river ice break-up patterns can also contribute to a better understanding of the impacts of climate change on river systems. As temperatures continue to warm, river ice break-up patterns are expected to change, potentially leading to more frequent and severe flooding events. As the results suggest, during the second 9-year period, there is a difference between the high latitude and mid-low-latitude stations and their behavior. Understanding the historical patterns of ice break-up can inform mitigation and adaptation efforts to address the impacts of climate change due to this resulted behavior.

The presence of the Torneträsk Lake behind the Abisko station could potentially affect the local microclimate and river ice break-up patterns. Lakes can have a significant impact on local climate and weather patterns (Long et al., 2007). They can affect temperature by storing and releasing heat, as well as by influencing local moisture levels (Croley, 1992). For example, during the winter, lakes can freeze over and create a layer of insulating ice that can prevent heat loss from the lake to the atmosphere. In the case of river ice break-up, the presence of a lake behind the Abisko station could potentially affect the timing and pattern of ice break-up in the Abisko station. The Torneträsk Lake is big enough to influence the temperature and water flow in the river. It could act as a heat sink (Newton & Mullan, 2021) and keep the surrounding area cooler than it would be (Fink et al., 2014). Similarly, the lake could affect water flow by acting as a buffer and changing the water flow rate into the river.

It is important to note that this study has some limitations, to keep the analysis consistent with available temperature data -as the most dominant factor on BUDs in our results-, the sample size was limited to only 18 years, which may not represent long-term trends. Regarding verification of BUD estimations, available SAR data were used. SAR data are sensitive to the surface properties (texture, roughness, geometry) in their backscatter values (Alijani et al., 2021). As the ice surface typically results in higher backscatter compared to liquid water, when the river is Frozen, we observe higher Backscatter in SAR data. This is due to the ice texture having a rougher surface compared to smooth water and can contain air bubbles and other inclusions that contribute to the radar signal's scattering (Murfitt et al., 2024; Palomaki & Sproles, 2022; Tian et al., 2015). The ice surface might also have snow cover, which can further influence the backscatter depending on the snow's moisture content, density, and structure (Murfitt et al., 2024; Pivot, 2012). Melting results in the formation of liquid water, which generally has a much lower radar backscatter than ice. Liquid water tends to absorb more of the radar signal and reflects less back to the satellite, which means lower backscatter values (Canisius et al., 2019). Once the river is entirely in a liquid state, the backscatter decreases significantly and can be tracked visually.

Due to some limitations, we faced uncertainties that needed to be verified by higher-resolution RADAR images. Several factors can introduce uncertainty in the interpretation of SAR backscatter values and the derived break-up event. The presence of physical obstacles along and around the river, sentinel-1 spatial resolution (30 m after de-speckling), as well as the narrow width of the river in certain areas, may result in year-to-year variations in the backscatter values. These variations can introduce some level of uncertainty in the break-up detection process for certain years and stations. Despite these potential uncertainties, the integration of RiTiCE's calculated BUD and Sentinel-1 SAR data provides visual validation regarding the break-up timings estimated by RiTiCE at some stations. The defined BUD in this study can be a unique definition of break-up and fills the gap in two ways, (1) lack of observed ice break-up data in some rivers (Dibike et al., 2021; White et al., 2007; Yang et al., 2020), and (2) inconsistent definition of break-up in different regions (Cooley & Pavelsky, 2016; Newton & Mullan, 2021; Prowse et al., 2007). This combination of methodologies offers a promising approach to studying and monitoring river ice dynamics and their temporal variations.

The observed ice break-up data on Abisko, Karesuvanto, and Kukkolankoski stations verified the RiTiCE BUD detection tool results. A significant correlation exists between the estimated BUDs by RiTiCE and observed BUDs in all stations (r = 0.6 – 0.8). However, it should be noted that the definition of break-up is considered arbitrary in different stations; for instance, in Kukkolankoski, the BUD is observed when a frozen raft in the river moves. In Övertorneå location (upstream of Kukkolankoski), a raft is also an indicator; the time when the raft passes the bridge over the river indicates the break-up time. There are some issues regarding these indicators. Occasionally, the ice in the River Tornionjoki may melt, allowing water to flow unrestricted in certain areas before the raft begins to move. This can result in an overestimation of the BUD. The raft in Övertorneå can also get stuck under the bridge, which occurred in 2010. There have also been sabotage attempts on the raft location (Persson, 2012). Therefore, the BUDs might correlate significantly, but there is a time lag (average of two weeks) between the estimated and observed BUDs due to different definitions of ice break-up. However, this does not oppose the aim of the study, which is to find the break-up patterns in different stations.

## Conclusion

This study covers the ice phenology of the River Tornionjoki basin and highlights the differences in ice break-up patterns across different latitudes. It is an attempt to fill the gap of data scarcity by applying RiTiCE tool to find the timing of break-up events in stations were there are no records of river ice break-up events, and to fill the gap in different definition of river ice break-up in different regions. The method is more developed by incorporating other hydrometeorological factors, such as longwave and shortwave irradiance data, wind speed, air temperature, and relative humidity. The results indicate that river ice tends to break up earlier in low and mid-latitude stations compared to high-latitude stations. Additionally, our research suggests that the timing of BUD and TS PCT varies between stations and is affected by local conditions such as morphology, width of river, water level, and other climatic factors that were not considered. Moreover, the study revealed that the break-up patterns in some stations, including Karesuvanto, Kallio, Kukkolankoski, and Abiskojokk, are less consistent and cover a broader range of values than other locations owing to the dominating influence of different variables on break-up events. The findings of this study have significant ecological and socio-economic implications as changes in ice phenology can affect river ecosystems, transportation, and hydropower production in some rivers. This study provides a better understanding of the dynamics of river ice cover at the basin scale and could be useful in developing effective strategies for managing and adapting to changing ice conditions in the River Tornionjoki basin.

## Data Availability

The data used in this study belongs to SYKE (Finnish Environment Institute) and SMHI (Swedish Meteorological and Hydrological Institute) and are publicly available upon registration, other variables were collected from NASA—Prediction of Worldwide Energy Resource data access viewer. The data used in all analysis can be obtained from the corresponding author upon reasonable request.

## Appendix

10.6084/m9.figshare.24421804.
